# Sex differences in clinical characteristics and risk factors for mortality among severe patients with COVID-19: a retrospective study

**DOI:** 10.18632/aging.103793

**Published:** 2020-10-13

**Authors:** Wating Su, Zhen Qiu, Lu Zhou, Jiabao Hou, Yafeng Wang, Fengnan Huang, Yi Zhang, Yifan Jia, Jun Zhou, Danyong Liu, Zhengyuan Xia, Zhong-Yuan Xia, Shaoqing Lei

**Affiliations:** 1 Department of Anesthesiology, Renmin Hospital of Wuhan University, Wuhan, China; 2 Department of Pain Medicine, Renmin Hospital of Wuhan University, Wuhan, China; 3 Department of Anesthesiology, Affiliated Hospital of Guangdong Medical University, Zhanjiang, Guangdong, China; 4 Department of Anesthesiology, The University of Hong Kong, Hong Kong, China

**Keywords:** COVID-19, sex difference, risk factors, mortality

## Abstract

The coronavirus disease 2019 (COVID-19) became a global pandemic. Males, compared to females, seem to be more susceptible to COVID-19, but related evidence is scarce, especially in severe patients. We explored sex differences in clinical characteristics and potential risk factors for mortality in severe COVID-19 patients. In this retrospective cohort study, we included all severe COVID-19 patients admitted to Eastern Renmin Hospital of Wuhan University, Wuhan, China, with a definitive clinical outcome as of Apr 10, 2020. Of the included 651 patients, 332 were male, and 319 were female. Males and females did not differ in age and underlying comorbidities. Males were more likely than females to report fever and develop serious complications, including acute respiratory distress syndrome, secondary infection, acute cardiac injury, coagulopathy, acute kidney injury and arrhythmia. Further, males had much higher mortality relative to females. Multivariable regression showed neutrophilia (odds ratio 6.845, 95% CI 1.227-38.192, *p*=0.028), thrombocytopenia (19.488, 3.030-25.335, *p*=0.002), hypersensitive troponin I greater than 0.04 pg/mL (6.058, 1.545-23.755, *p*=0.010), and procalcitonin greater than 0.1 ng/mL (6.350, 1.396-28.882, *p*=0.017) on admission were associated with in-hospital death. With either of these risk factors, the cumulative survival rate was relatively lower in males than in females. In conclusion, males are more likely than females to develop serious complications and progress to death. The potential risk factors of neutrophilia, thrombocytopenia, hypersensitive troponin I greater than 0.04 pg/mL and procalcitonin more than 0.1 ng/mL may help clinicians to identify patients with poor outcomes at an early stage, especially in males.

## INTRODUCTION

The ongoing coronavirus disease 2019 (COVID-19) caused by the severe acute respiratory syndrome coronavirus 2 (SARS-CoV-2) infection has rapidly grown into a global pandemic. As of May 2, 2020, a total of 3,267,184 laboratory-confirmed cases have been identified around the world, and nearly 230,000 patients have died of this pandemic according to the report from the WHO [1]. In addition to the common clinical manifestations of respiratory failure caused by COVID-19, sex differences during COVID-19 disease progression have attracted increasing attention [2]. Several studies suggested old males with comorbidities were more likely than females to be infected with COVID-19 [3, 4], and had a high likelihood of progressing to severe illness [5, 6]. Epidemiological analysis of cases and deaths had identified numerous risk factors for mortality from complications caused by COVID-19. Specifically, the risk of serious complications and mortality was highly age-dependent [7]. However, at all ages, males seemed to be more likely than females to develop serious complications and progress to death [7–9]. It has long been known that a male bias is observed in total mortality rates [10], and in death owing to specific diseases [11]. However, currently, no study has yet comprehensively investigated sex differences in clinical characteristics, mortality from serious complications, and the risk factors of in-hospital death in males versus females in patients with COVID-19, especially in severely ill patients.

In this study, we presented the details of all severe patients with COVID-19, who were admitted to Eastern Renmin Hospital of Wuhan University, a designated hospital in Wuhan, China, and who had a definitive clinical outcome as of Apr 10, 2020. We aimed to explore sex differences in clinical characteristics and potential risk factors for in-hospital mortality from complications arising from COVID-19.

## RESULTS

### Demographics and characteristics

1530 patients with confirmed SARS-CoV-2 detection were admitted to Eastern Renmin Hospital of Wuhan University as of Apr 10, 2020. After excluding 776 patients that were still hospitalized or not diagnosed as severe illness as of Apr 10, 2020, as well as 103 inpatients without available key information in their medical records, we included 651 severe patients in the final analysis. Among these patients, the median age was 62 years (IQR, 49-71), ranging from 18 years to 98 years, 287 (44.1%) patients were elderly, and 332 (51.0%) patients were male (Table 1). All these patients were Wuhan citizen, of whom 5.2% patients were current smokers, and 35.8% patient had a clear contact history with confirmed COVID-19 patients. They all presented bilateral involvements of typical chest computerized tomography manifestations of COVID-19 pneumonia on admission. 274 of 651 (42.1%) patients had comorbidities. The most common comorbidity was hypertension (183 [28.1%]), followed by diabetes (116 [17.8%]), cardiovascular disease (83 [12.7%]), chronic lung disease (31 [4.8%]), cerebrovascular disease (28 [4.3%]), chronic kidney disease (20 [3.1%]), chronic liver disease (20 [3.1%]), and malignancy (16 [2.5%]). The most common symptoms on hospital admission were fever (540 [82.9%]), dry cough (338 [51.9], fatigue (217 [33.3%]), dyspnea (207 [31.8%]), expectoration (133 [20.4%]), and anorexia (97 [14.9%]). Dizziness (25 [3.8]), headache (23 [3.5%]), nausea (18 [2.8%]), vomiting (18 [2.8%]), abdominal pain (6 [0.9%]), and hemoptysis (4 [0.6%]) were rare (Table 1). Compared to the female patients, male patients were more likely to report clear exposure history (*P* =0.003) and have fever (*P* =0.0046).

**Table 1:** Baseline characteristics between female and male patients.

	**Total (n = 651)**	**Male (n = 332)**	**Female (n = 319)**	**P value**
Age, years	62 (49-71)	62 (49-73)	62 (49-70)	0.32
≥ 65	287 (44.1)	148 (44.6)	139 (43.6)	0.80
< 65	364 (55.9)	184 (55.4)	180 (56.4)	..
Exposure history*	233 (35.8)	137 (41.3)	96 (30.9)	0.003
Current smoker	34 (5.2)	21 (6.3)	13 (4.1)	0.22
**Comorbidity**	274 (42.1)	150 (54.2)	124 (38.9)	0.10
Hypertension	183 (28.1)	102 (30.7)	81 (25.4)	0.13
Diabetes	116 (17.8)	60 (18.1)	56 (17.6)	0.86
Cardiovascular disease	83 (12.7)	37 (11.1)	46 (14.4)	0.21
Chronic lung disease	31 (4.8)	19 (5.7)	12 (3.8)	0.24
Cerebrovascular disease	28 (4.3)	15 (45.2)	13 (40.8)	0.78
Chronic kidney disease	20 (3.1)	12 (4.5)	8 (2.5)	0.16
Chronic liver disease	20 (3.1)	14 (4.2)	6 (1.9)	0.084
Malignancy	16 (2.5)	7 (2.1)	9 (2.8)	0.56
**Signs and symptoms**				
Fever	540 (82.9)	289 (87.0)	251 (78.7)	0.0046
Dry cough	338 (51.9)	172 (51.8)	166 (52.0)	0.95
Fatigue	217 (33.3)	110 (33.1)	107 (33.5)	0.91
Dyspnea	207 (31.8)	109 (32.8)	98 (30.7)	0.56
Expectoration	133 (20.4)	77 (23.2)	56 (17.6)	0.075
Anorexia	97 (14.9)	57 (17.2)	40 (12.5)	0.10
Diarrhea	63 (9.7)	30 (9.0)	33 (10.3)	0.57
Myalgia	47 (7.2)	24 (72.3)	23 (72.1)	> 0.99
Pharyngalgia	42 (6.5)	19 (5.7)	23 (7.2)	0.44
Dizziness	25 (3.8)	13 (3.9)	12 (3.8)	0.92
Headache	23 (3.5)	12 (3.6)	11(3.5)	0.91
Nausea	18 (2.8)	12 (3.6)	6 (1.9)	0.18
Vomiting	18 (2.8)	8 (2.4)	10 (3.1)	0.57
Abdominal pain	6 (0.9)	1 (0.3)	5 (1.6)	0.091
Hemoptysis	4 (0.6)	2 (0.6)	2 (0.6)	0.97

*These patients had clear contact history with the confirmed COVID-19 patients.

*P* < 0.05 was considered statistically significant.

### Laboratory findings

Laboratory values on admission were summarized in Table 2. The most common hematologic abnormalities were lymphocytopenia (327 [50.2%]), neutrophilia (153 [23.5%]), mononucleosis (144 [22.1%]), and thrombocytopenia (63 [9.7%]). Most patients presented decreased T-lymphocyte, including CD3 (226 of 335 [67.5%]), CD4 (215 of 335 [64.2%]), CD8 (204 of 335 [60.9%]), CD16+56 (121 of 335 [36.1%]), and CD19 (95 of 335 [28.4%]). Most patients had elevated cardiac indices, including lactate dehydrogenase (269 of 427 [63.0%]), N-terminal pro-B-type natriuretic (102 of 324 [31.5%]), myoglobin (83 of 355 [23.4%]), Hs-TnI (95 of 432 [22.0%]), creatine kinase-MB (78 of 466 [16.7%]), and creatine kinase (49 of 432 [11.3%]). Coagulation abnormalities were also obvious: 340 of 544 (62.5%) patients had raised D-dimer, 111 of 431 (25.8%) had prolonged activated partial thromboplastin time, and 83 of 434 (19.1%) had prolonged prothrombin time. More than half of patients had COVID-19 related inflammation indicated by elevated C-reactive protein (277 of 430 [64.4%]) and procalcitonin (164 of 398 [41.2%]). Some patients demonstrated liver injury with elevated aspartate aminotransferase (191 of 607 [31.5%]), alanine aminotransferase (153 of 607 [25.2%]), and total bilirubin (35 of 450 [7.8%]). Kidney injury indicated by elevated blood urea nitrogen (115 of 515 [22.3%]) and creatinine (161 of 513 [31.4%]) was also considerable.

**Table 2:** Laboratory findings between female and male patients on admission.

	**Total (n = 651)**	**Male (n = 332)**	**Female (n = 319)**	**P value**
**Hematologic**				
White blood cell count, ×10^9^/L	5.7 (4.4-7.8)	5.8 (4.6-8.0)	5.7 (4.4-7.5)	0.15
< 3.5	70 (10.8)	33 (9.9)	37 (11.6)	0.49
3.5-9.5	498 (76.5)	246 (74.1)	252 (79.0)	0.14
> 9.5	83 (12.7)	53 (16.0)	30 (9.4)	0.012
Neutrophil count, ×10^9^/L	3.8 (2.7-6.1)	4.0 (2.8-6.5)	3.6 (2.6-5.5)	0.026
> 6.3	153 (23.5)	89 (26.8)	64 (20.1)	0.042
Lymphocyte count, ×10^9^/L	1.1 (0.7-1.6)	0.9 (0.6-1.5)	1.2 (0.8-1.7)	<0.001
< 1.1	327 (50.2)	190 (57.2)	137 (42.9)	<0.001
Monocyte count, ×10^9^/L	0.42 (0.29-0.57)	0.44 (0.28-0.60)	0.40 (0.29-0.54)	0.25
> 0.6	144 (22.1)	82 (24.7)	62 (19.4)	0.106
Platelet count, ×10^9^/L	205 (149-268)	195 (142-253)	213 (158-288)	0.001
< 100	63 (9.7)	38 (11.4)	25 (7.8)	0.12
CD3, /μl	509 (298-856)	464 (272-727)	568 (355-931)	0.009
<723	226/335 (67.5)	140/187 (74.9)	86/148 (58.1)	0.0012
CD4, /μl	297 (183-529)	268 (162-439)	357 (193-590)	0.003
< 404	215/335 (64.2)	133/187 (71.1)	82/148 (55.4)	0.0029
CD8, /μl	175 (95-295)	161 (86-268)	200 (115-335)	0.01
< 220	204/335 (60.9)	124/187 (66.3)	80/148 (54.1)	0.022
CD4/CD8	1.82 (1.22-2.59)	1.82 (1.17-2.63)	1.85 (1.32-2.53)	0.81
CD19, /μl	122 (73-189)	116 (64-169)	131 (79-225)	0.013
< 80	95/335 (28.4)	57/187 (30.5)	38/148 (25.7)	0.33
CD16+56, /μl	108 (67-170)	112 (71-175)	99 (67-151)	0.13
< 84	121/335 (36.1)	59/187 (31.6)	62/148 (41.9)	0.050
**Biochemical**				
ALT, U/L	25 (17-41)	29 (21-47)	21 (15-33)	<0.001
> 40	153/607 (25.2)	99/309 (32.0)	54/298 (18.1)	<0.001
AST, U/L	27 (19-40)	30 (22-47)	23 (17-34)	<0.001
> 35	191/607 (31.5)	121/310 (39.0)	70/297 (23.6)	<0.001
Total bilirubin, uM	10.7 (7.5-15.5)	12.0 (98.4-16.2)	9.6 (7.2-14.9)	0.001
> 23	35/450 (7.8)	18/239 (7.5)	17/211 (8.1)	0.84
Blood urea nitrogen, mM	5.0 (3.8-7.3)	5.4 (4.4-7.7)	4.3 (3.4-6.2)	<0.001
> 7.5	115/515 (22.3)	73/270 (27.0)	42/245 (17.1)	0.0071
Creatinine, uM	64 (52-78.)	72 (61-84)	55 (46-65)	<0.001
> 73	161/513 (31.4)	120/269 (44.6)	41/244 (16.8)	<0.001
LDH, U/L	285 (217-419)	308 (242-447)	256 (202-366)	<0.001
> 250	269/427 (63.0)	164/228 (71.9)	105/199 (52.8)	<0.001
Creatine kinase, U/L	66 (40-126)	85 (49-155)	51 (35-87)	<0.001
> 310	49/432 (11.3)	34/231(14.7)	15/201 (7.5)	0.018
Myoglobin, ug/L	53 (30-104)	67 (39-122)	36 (25-78)	<0.001
> 110	83/355 (23.4)	54/195 (27.7)	29/160 (18.1)	0.034
CK-MB, ng/mL	1.26 (0.77-2.97)	1.33 (0.82-3.40)	1.21 (0.67-2.58)	0.10
> 5	78/466 (16.7)	39/238 (16.4)	39/228 (17.1)	0.84
Hs-TnI, pg/mL	0.01 (0.006-0.288)	0.01 (0.006-0.048)	0.01 (0.009-0.021)	0.19
> 0.04	95/432 (22.0)	60/225 (26.7)	35/207 (16.9)	0.014
Pro-BNP, pg/mL	185.6 (60.4-569.5)	184.1 (54.7-535.2)	183.0 (68.6-605.8)	0.71
> 450	102/324 (31.5)	53/174 (30.5)	49/150 (32.7)	0.67
CRP, mg/L	25.4 (5.0-76.6)	34.8 (9.9-87.7)	12.0 (5.0-61.5)	<0.001
> 10	277/430 (64.4)	174/233 (74.7)	103/197 (52.3)	<0.001
Procalcitonin, ng/mL	0.08 (0.04-0.21)	0.10 (0.05-0.25)	0.05 (0.03-0.15)	<0.001
> 0.1	164/398 (41.2)	106/213(49.8)	58/185 (31.4)	<0.001
**Coagulation function**				
PT, s	12.1 (11.5-12.8)	12.3 (11.6-12.9)	11.9 (11.2-12.7)	0.01
>13	83/434 (19.1)	54/232 (23.3)	29/202 (14.4)	0.018
APTT, s	28.2 (25.9-31.5)	28.8 (26.5-32.6)	27.5 (25.2-30.5)	<0.001
> 31.3	111/431 (25.8)	75/229 (32.8)	36/202 (17.8)	<0.001
D-dimer, mg/L	0.79 (0.39-2.48)	0.80 (0.40-3.34)	0.78 (0.39-2.08)	0.17
> 0.55	340/544 (62.5)	181/279 (64.9)	159/265 (60.0)	0.24
**Arterial blood gas analysis**			
PH	7.42 (7.38-7.46)	7.42 (7.38-7.46)	7.42 (7.38-7.47)	0.57
PaO_2_, mmHg	74.0 (55.0-98.2)	70.0 (52.3-94.0)	76.5 (58.0-99.8)	0.083
< 80	154/274 (56.2)	92/150 (61.3)	62/124 (50.0)	0.060
PaCO_2_, mmHg	39 (35-44)	38 (34-44)	40 (35-44)	0.56
Glucose, mmol/L	7.4 (5.8-10.0)	7.2 (5.7-10.0)	7.5 (6.1-9.9)	0.50
> 6.1	183/264 (69.3)	96/146 (65.8)	87/118 (73.7)	0.16
Lac	2.2 (1.7-2.8)	2.1 (1.7-2.7)	1.70 (2.3-2.9)	0.55

Abbreviations: AST, aspartate aminotransferase; ALT, alanine aminotransferase; APPT, activated partial thromboplastin time; CK-MB, creatine kinase myocardial isoenzyme; CRP, C-reactive protein; Hs-TnI, high-sensitivity cardiac troponin I; LDH, lactate dehydrogenase; NT-proBNP, N-terminal pro-B-type natriuretic peptide; PaCO_2_, arterial partial pressure of carbon dioxide; PaO_2_, arterial oxygen partial pressure; PT, prothrombin time.

*P* < 0.05 was considered statistically significant.

Compared to female patients, male patients showed significantly higher neutrophil count (*P* = 0.026), lower counts of lymphocyte (*P* < 0.001) and platelet (*P* = 0.001) (Table 2). Male patients also presented significantly lower counts of CD3 (*P* = 0.009), CD4 (*P* = 0.003), CD8 (*P* = 0.01) and CD19 (*P* = 0.013) T-cells as compared with female patients. Additionally, male patients had higher levels of alanine aminotransferase (*P* < 0.001) and aspartate aminotransferase (*P* < 0.001), and longer prothrombin time (*P* = 0.01) and activated partial thromboplastin time (*P* < 0.001). Total bilirubin, urea nitrogen, creatinine, lactate dehydrogenase, creatine kinase, myoglobin, C-reactive protein, and procalcitonin were also significantly higher in male patients than female patients (all *P* < 0.05) (Table 2).

### Treatments, complications and clinical outcomes

Among the 651 patients, 95.6% received antiviral medications (eg, lopinavir, ritonavir), 78.3% patients received antibiotics, 34.1% received glucocorticoid, and only 2.0% received continuous renal replacement therapy (Table 3). 336 of 651 patients (51.6%) patients received high-flow nasal cannula oxygen therapy, 62 (9.5%) patients received noninvasive mechanical ventilation, and 45 (6.9%) patients required invasive mechanical ventilation, of whom 3 received extracorporeal membrane oxygenation as rescue therapy. ARDS (266 [40.8%]) was the most common complication, followed by secondary infection (103 [15.8%]), acute cardiac injury (93 [14.3%]), coagulopathy (90 [13.8%]), septic shock (63 [9.7%]), acute kidney injury (58 [8.9%]) and arrhythmia (18 [2.8%]). The media time from first symptoms to hospital admission was 11 days (IQR, 6-17), and 11.5 days (IQR, 4.8-18.5) to ARDS. The median length of hospital stay was 20 days (IQR, 9-30).

**Table 3:** Treatments, complications, and outcomes between female and male patients.

	**Total (n=651)**	**Male (n=332)**	**Female (n=319)**	**P value**
**Treatments**				
Antiviral therapy	609 (95.6)	312 (94.0)	297 (93.1)	0.77
Antibiotic therapy	510 (78.3)	264 (79.5)	246 (77.1)	0.52
Glucocorticoid therapy	222 (34.1)	133 (40.1)	89 (27.9)	0.0014
CRRT	13 (2.0)	8 (2.4)	5 (1.6)	0.63
High-flow nasal cannula oxygen therapy	336 (51.6)	175 (52.7)	161 (50.5)	0.61
Noninvasive ventilation	62 (9.5)	35 (10.5)	27 (8.5)	0.44
Invasive mechanical ventilation	45 (6.9)	27 (8.1)	18 (5.6)	0.27
ECMO	3 (0.5)	3 (0.9)	0 (0)	0.074
**Complications**				
ARDS	266 (40.8)	160 (48.2)	106 (33.2)	< 0.001
Secondary infection	103 (15.8)	72 (21.7)	31 (9.7)	< 0.001
Acute cardiac injury	93 (14.3)	60 (18.1)	33 (10.3)	0.0049
Coagulopathy	90 (13.8)	58 (17.5)	32 (10.0)	0.0084
Septic shock	63 (9.7)	36 (10.8)	27 (8.5)	0.30
Acute kidney injury	58 (8.9)	38 (11.4)	20 (6.2)	0.021
Arrhythmia	18 (2.8)	14 (4.2)	4 (1.3)	0.039
The time from first symptom to hospital admission, days	11 (6-17)	11 (6-16)	11 (6-18)	0.82
The time from first symptom to ARDS, days	11.5 (4.8-18.5)	14.5 (6.5-25.5)	10.5 (3.3-18.5)	0.38
Length of hospital stay, days	20 (9-30)	20 (9-30)	19 (9-30)	0.64
**Prognosis**				
Death	147 (22.6)	90 (27.1)	57 (17.9)	0.0048
Survival	504 (77.4)	242 (72.9)	262 (82.1)	0.006

Abbreviations: ARDS, acute respiratory distress syndrome; CRRT, continuous renal replacement therapy ECMO, extracorporeal membrane oxygenation;

*P* < 0.05 was considered statistically significant.

Male patients were more likely to receive glucocorticoid therapy (133 [40.1%] vs 89 [27.9%]) as compared with female patients. The frequencies of ARDS (160 [48.2] vs 106 [33.2]), secondary infection (72 [21.7%] vs 31 [9.7%]), acute cardiac injury (60 [18.1%] vs 33 [10.3]), coagulopathy (58 [17.5%] vs 32 [10.0%]), acute kidney injury (38 [11.2%] vs 20 [6.2%]) and arrhythmia (14 [4.2%] vs 4 [1.3%]) were significantly higher than female patients (all *P* < 0.05). Of note, the mortality in male patients was much higher than female patients (*P* < 0.05).

### Risk factors for in-hospital death

As shown in Table 4, univariable logistic regression analysis revealed that sex, old age, leucocytosis, neutrophilia, lymphocytopenia, thrombocytopenia, CD3, CD4 and CD8 T-cells, alanine aminotransferase, aspartate aminotransferase, total bilirubin, blood urea nitrogen, creatinine, lactate dehydrogenase, creatine kinase, myoglobin, Hs-TnI, C-reactive protein, prothrombin time, activated partial thromboplastin time, and procalcitonin were associated with in-hospital death in severe patients with COVID-19. Multivariable logistic regression model further showed that neutrophilia (OR 6.845, 95% CI 1.227-38.192, *p* = 0.028), thrombocytopenia (OR 19.488, 95%CI 3.030-25.335, *p* = 0.002), Hs-TnI greater than 0.04 pg/mL (OR 6.058, 95%CI 1.545-23.755, *p* = 0.010), and procalcitonin greater than 0.1 ng/mL (OR 6.350, 95%CI 1.396-28.882, *p* = 0.017) on admission were associated with the incidence of in-hospital death (Table 4).

**Table 4:** Logistic regression analysis on risk factors associated with in-hospital death.

**Variables**	**Univariable OR (95% CI)**	***P* value**	**Multivariable OR (95% CI)**	***P* value**
Sex, male	1.709 (1.175-2.487)	0.005	0.697 (0.207-2.349)	0.561
Age ≥ 65 years	6.016 (3.947-9.170)	<0.001	3.776 (0.900-15.848)	0.069
White blood cell count > 9.5×109/L	8.321 (5.023-13.786)	<0.001	2.972 (0.504-17.530)	0.229
Neutrophil > 6.3×109/L	7.577 (5.010-11.462)	<0.001	6.845 (1.227-38.192)	0.028
Lymphocyte count				
< 1.1×109/L	10.518 (6.132-18.040)	<0.001	0.471 (0.061-3.615)	0.469
Platelet count				
< 100 ×109/L	4.787 (2.753-8.325)	<0.001	19.488 (3.030-125.335)	0.002
CD3, ≤ 723/μL	14.107 (5.010-39.722)	<0.001	5.716 (0.344-95.076)	0.224
CD4, ≤ 404/μL	35.664 (8.581-148.222)	<0.001	0.876 (0.050-15.345)	0.928
CD8, ≤ 220/μL	5.294 (2.737-10.241)	<0.001		
ALT > 40 U/L	1.530 (1.002-2.337)	0.049		
AST > 35 U/L	5.270 (3.497-7.942)	<0.001	1.235 (0.332-4.588)	0.753
Blood urea nitrogen > 7.5 mM	10.339 (6.443-16.591)	<0.001	3.751 (0.964-14.593)	0.057
Creatinine > 73 uM	3.540 (2.326-5.387)	<0.001		
LDH > 250 U/L	11.810 (5.777-24.145)	<0.001	3.401 (0.592-19.545)	0.170
Creatine kinase > 310 U/L	4.994 (2.685-9.290)	<0.001		
Myoglobin > 110 ug/L	10.558 (6.021-18.516)	<0.001	1.569 (0.406-6.058)	0.513
Hs-TnI > 0.04 pg/mL	13.063 (7.689-22.194)	<0.001	6.058 (1.545-23.755)	0.010
CRP > 10 mg/L	37.703 (9.139-155.542)	<0.001	3.638 (0.195-67.827)	0.387
PT > 13 s	4.800 (2.894-7.961)	<0.001	0.709 (0.163-3.076)	0.646
APTT > 31.3 s	1.721 (1.083-2.737)	0.022		
Procalcitonin > 0.1 ng/mL	8.306 (5.017-13.751)	<0.001	6.350 (1.396-28.882)	0.017

Abbreviations: AST, aspartate aminotransferase; ALT, alanine aminotransferase; APPT, activated partial thromboplastin time; CRP, C-reactive protein; Hs-TnI, high-sensitivity cardiac troponin I; LDH, lactate dehydrogenase; PT, prothrombin time.

*P* < 0.05 was considered statistically significant.

**Figure 1:**
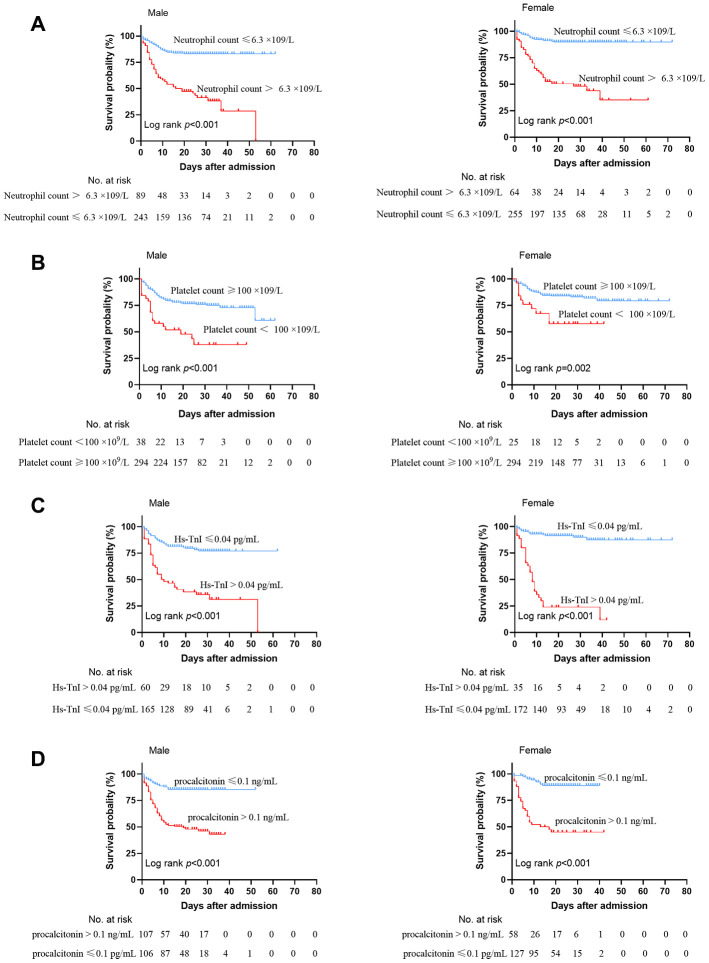
**Kaplan-Meier survival curves for mortality between male and female patients.** (**A**) Neutrophil count. (**B**) Platelet count. (**C**) Hs-TnI ≤ 0.04 pg/mL. (**D**) procalcitonin ≤ 0.1 ng/mL.

The Kaplan-Meier survival curve showed that the cumulative survival rate during hospitalization was significantly lower in patients with neutrophilia, thrombocytopenia, Hs-TnI greater than 0.04 pg/mL or procalcitonin greater than 0.1 ng/mL as compared with the corresponding normal indices both in male and female patients (Figure 1). Compared to female patients, the cumulative survival rate of male patients with either of these risk factors was relatively lower. Male patients were less tolerant to neutrophilia, thrombocytopenia, Hs-TnI greater than 0.04 pg/mL and procalcitonin more than 0.1 ng/mL relative to female patients.

## DISCUSSION

This retrospective cohort study, to our knowledge, is the first study to present sex differences in clinical characteristics among severe patients with COVID-19. We also identified sex differences in potential risk factors for the incidence of in-hospital death. Specifically, we found neutrophilia, thrombocytopenia, hs-TnI greater than 0.04 pg/mL and procalcitonin greater than 0.1 ng/mL on admission were associated with higher odds of in-hospital death in males.

In the current study, males are more likely than females to develop serious complications after severe COVID-19 infection, resulting in a higher mortality in males than in females, which is consistent with the previous findings that the death rate of males was much higher than females at all ages [7–9]. A previous study had documented risk factors of in-hospital mortality in adult patients with COVID-19 and pre-existing comorbidities (eg, cardiovascular disease, diabetes, hypertension) [12]. However, our study showed no significant difference in age and pre-existing comorbidities between male and female patients with severe COVID-19, suggesting sex differences after virus infection might be more important for adverse outcomes.

It was well demonstrated that high levels of circulating inflammatory cytokines correlated with the severity of illness and mortality in COVID-19 patients [5, 13]. Also, dysregulated immune responses were shown to play an important role in COVID-19 pathogenesis [14, 15], and functional exhaustion of antiviral lymphocytes occurred in severely ill patients with COVID-19 [16]. Of note, males, compared to females, were more susceptible to viral infections based on a different innate immunity related to sex chromosomes that immune genes were more expressed on the X chromosome [2]. In this cohort, we demonstrated significantly higher levels of infection-related indices and lower levels of immune cells in male patients with severe COVID-19 relative to females. In particular, neutrophilia and procalcitonin greater than 0.1 ng/mL were found to be independent predictors of in-hospital death, especially in males with severe COVID-19.

Angiotensin converting enzyme 2 (ACE2) is the entry receptor for SARS-CoV-2 infection. Additionally, ACE2 has important immune modulation roles in controlling excess inflammation in the presence of danger signals [17]. ACE2 gene is located on the X chromosome, such that females have two copies of the ACE2 gene, compared to a single copy in males. However, in COVID-19 infections, ACE2 expression levels have no significant differences between males and females, or between younger and older persons in any tissue [18]. Intriguingly, consistent with our present study, COVID-19 mostly affected males [3, 4], and the mortality was much higher in males than in females [7–9]. These may be explained by the fact that ACE2 expression levels are positively and negatively associated with immune signatures in males and females, respectively [18]. It has been reported that circulating ACE2 levels are sex dependent in patients, being 50% higher in males than in females in heart failure [19]. Whereas circulating levels do not directly reflect tissue levels, ACE2 is released as part of tissue response to stress thereby up-regulating ACE2 function. In this study, the frequency of increased Hs-TnI (an indicator of myocardial injury) during hospitalization was much higher in males as compared with females, suggesting that males, are more susceptible to myocardial injury or even heart failure after COVID-19 infection. In addition, we suggest Hs-TnI greater than 0.04 pg/mL is a “pro-death” factor that precipitates acute cardiac injury into death, which is in line with the previous finding that cardiac injury is associated with higher risk of in-hospital mortality [20, 21].

Previous study showed about 90% of inpatients with community-acquired pneumonia had coagulation activity, indicated by elevated d-dimer concentrations [22]. Our study showed elevated d-dimer in COVID-19 patients in more than half of the severe COVID-19 patients, but it did not differ between males and females. However, we showed higher frequency of prolonged prothrombin time and activated partial thromboplastin time in males. Thrombocytopenia was suggested to be associated with the severity of illness after COVID-19 infection [23]. In this study, we found platelet count were significantly lower in males than in females. Our present study further identified thrombocytopenia as an independent predictor of in-hospital death, especially in males.

There were several limitations in the present study. First, it was a single-center study, which may affect the interpretation of our findings. Second, some patients developed secondary infection, which may affect the outcome of the immune response. Third, as a clinical retrospective, there is a lack of specific mechanisms of gender differences on immune function and organ damage in severe COVID-19 patients. Thus, it is necessary to collect data from a larger cohort, especially different races and multiple centers, to further confirm sex differences in immunity, vital organ damage, and mortality.

In conclusion, males are more likely than females to develop serious complications and progress to death mainly due to sex difference in immune response. We found neutrophilia, thrombocytopenia, Hs-TnI greater than 0.04 pg/mL and procalcitonin more than 0.1 ng/mL were independent risk factor for in-hospital death, especially in male patients with severe COVID-19. These potential risk factors are helpful for clinicians to identify patients with poor outcomes at an early stage.

## MATERIALS AND METHODS

### Study design and participants

This retrospective cohort study was conducted at Eastern Renmin Hospital of Wuhan University, Wuhan, China, which was an assigned hospital to treat COVID-19 by the government. All patients who were diagnosed with COVID-19 according to WHO interim guidance [24] were screened from Jan 25, 2020 to Apr 10, 2020. We included all severe patients with COVID-19 in this study. Severe patients were those who meet any of the following criteria according to the Chinese management guideline for COVID-19 (version 7.0) [25]: 1) shortness of breath, respiratory frequency ≥ 30/min; 2) blood oxygen saturation ≤ 93%; 3) the ratio of arterial partial pressure of oxygen (PaO2) to fraction of inspired oxygen (FiO2) ≤ 300 mmHg; 4) chest CT imaging showing lung infiltrates > 50% within 24 to 48 hours.

This study was reviewed and approved by the Ethics Commission of Renmin Hospital of Wuhan University (approval number WDRY2020-K118). Written informed consent was waived by the designated hospital due to the emerging infectious disease.

### Data collection

The demographic characteristics (age, sex, exposure history), underlying comorbidities (eg, hypertension, diabetes, cardiovascular disease), clinical symptoms (eg, fever, dry cough, dyspnea), laboratory findings (eg, hematologic, biochemical, coagulation function), treatments (eg, antiviral, antibiotic, glucocorticoid therapy), complications (eg, acute respiratory distress syndrome [ARDS], secondary infection, acute cardiac injury), and outcomes (discharge and mortality) were collected from electronic medical records by three investigators. Another two investigators independently reviewed all the data to verify data accuracy.

### Outcomes

The primary outcomes were in-hospital mortality among male and female patients with COVID-19. The secondary outcomes included incidence of ARDS, acute cardiac injury, secondary infection, coagulopathy and acute kidney injury.

ARDS was diagnosed as acute-onset hypoxemia with bilateral pulmonary opacities on chest imaging which was not fully explained by other disease according to the Berlin Definition [26]. Secondary infection was defined if patients showed clinical symptoms or signs of bacteremia and a positive culture of a new pathogen was obtained from sputum or blood samples after admission [25]. Acute cardiac injury was defined when the blood concentration of cardiac biomarkers (eg, hypersensitive troponin I, Hs-TnI) was above the 99^th^ percentile reference limit or new abnormalities presented in electrocardiography and echocardiography [5]. Coagulopathy was defined as a 3-second extension of prothrombin time or a 5-second extension of activated partial thromboplastin time [12]. Sepsis was diagnosed according to the 2016 Third International Consensus Definition for Sepsis and Septic Shock [27]. Acute kidney injury was defined according to the Kidney Disease Improving Global Outcomes (KDIGO) criteria [28].

### Statistical analysis

Continuous variables were expressed as median (interquartile range, IQR), and compared by Mann-Whitney U test. Categorical variables were expressed as number (%), and compared by χ² test or Fisher’s exact test between male and female patients. To explore the risk factors associated with in-hospital mortality, univariable and multivariable binary logistic regression models were used. The Kaplan-Meier curve was performed with log-rank test for in-hospital mortality among female and male patients. A two-sided *p* value less than 0.05 was considered statistically significant. Data analysis was conducted with SPSS software (version 22.0). Statistical charts were generated by Graphpad Prism 8.0.
